# Bio-Based Deproteinized Natural Rubber/Alginate/Poly(vinyl alcohol) Films with Ca^2+^-Mediated and Tannic Acid-Induced Crosslinked Networks for Biodegradable Adhesive Materials

**DOI:** 10.3390/polym18202536

**Published:** 2026-10-15

**Authors:** Chaiwat Ruksakulpiwat, Supharat Inphonlek, Yupaporn Ruksakulpiwat

**Affiliations:** 1 School of Polymer Engineering, Institute of Engineering, Suranaree University of Technology, Nakhon Ratchasima 30000, Thailand; charuk@sut.ac.th (C.R.); supharat.inph@gmail.com (S.I.); 2 Research Center for Biocomposite Materials for Medical Industry and Agricultural and Food Industry, Suranaree University of Technology, Nakhon Ratchasima 30000, Thailand; 3 Institute of Research and Development, Suranaree University of Technology, Nakhon Ratchasima 30000, Thailand

**Keywords:** natural rubber composites, tannic acid crosslinking, bioadhesive materials, biodegradable polymer films

## Abstract

In this study, biodegradable adhesive composite films based on deproteinized natural rubber/alginate/poly(vinyl alcohol) (DPNR/ALG/PVA) were developed through Ca^2+^-mediated ionic crosslinking and tannic acid (TA)-induced hydrogen bonding interactions. DPNR/ALG/PVA mixtures were ionically crosslinked through ALG-Ca^2+^ interactions, cast into films, and subsequently immersed in TA-containing ethanol/water cosolvent systems. Treatment in a 75:25 ethanol/water solution at a PVA/TA weight ratio of 1:7 for 12 h produced stable films with a high gel fraction. The TA-treated DPNR/ALG3/PVA40 film exhibited gel fraction of 91.01 ± 0.79%, higher than that of the untreated film (71.22 ± 1.62%), indicating improved network stability after TA treatment. FTIR analysis confirmed the incorporation of ALG and PVA within the DPNR matrix and suggested the formation of interactions involving TA. Increasing ALG content from 1 to 5 phr enhanced swelling capacity, reaching 241.81 ± 22.30%, whereas increasing PVA content (20–80 phr) improved tensile strength from 3.51 ± 0.10 to 6.39 ± 0.43 MPa. Moreover, the PVA-TA network improved adhesive performance, increasing lap shear strength from 1.23 ± 0.20 to 1.83 ± 0.12 MPa and peel strength from 70.37 ± 15.10 to 280.94 ± 68.45 N/m. Soil burial tests revealed the biodegradability of composite films, with no recoverable residue detected after 180 days. These results demonstrate the potential of the developed composites as biodegradable adhesive materials for biomedical applications.

## 1. Introduction

**Figure 1:**
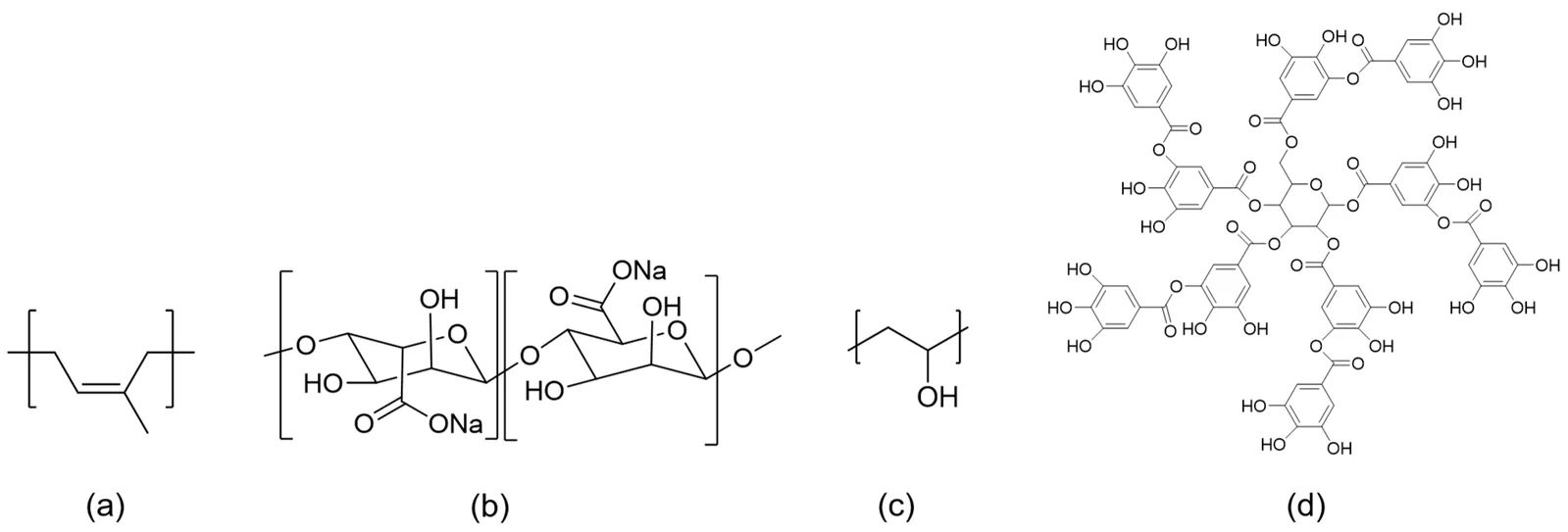
Chemical structure of main components used in this work (**a**) DPNR; (**b**) Sodium alginate; (**c**) Polyvinyl alcohol; (**d**) Tannic acid.

Flexible and adhesive biopolymer films have attracted considerable attention for biomedical and sustainable material applications, particularly in wound dressings, wearable devices, and skin-contact adhesive materials [1,2,3]. Natural polymer-based materials are increasingly recognized as promising candidates due to their biodegradability, biocompatibility, and renewability. Among these materials, natural rubber (NR) is an elastomeric biopolymer with excellent elasticity, toughness, and resilience, making it an attractive matrix for flexible bio-based composites. Deproteinized natural rubber (DPNR, Figure 1a) is particularly advantageous because the removal of allergenic proteins significantly improves biocompatibility [4,5] while preserving the physical and mechanical properties of NR. However, DPNR is intrinsically hydrophobic and contains limited functional groups, resulting in poor water absorption, low bioactivity, and insufficient interfacial interactions in aqueous or biological environments.

The incorporation of hydrophilic components into the rubber matrix is an effective approach for imparting additional functionalities, including enhanced water absorption and bioactivity. Polysaccharides such as alginate (ALG, Figure 1b) and synthetic biocompatible polymers such as poly(vinyl alcohol) (PVA, Figure 1c) have been widely used in hydrogel and film systems because of their excellent hydrophilicity, film-forming ability, and biocompatibility [6,7,8]. However, the intrinsic hydrophilic nature of these polymers, while beneficial for water absorption and bioactivity, renders them prone to swelling, dissolution, or structural disintegration upon prolonged exposure to water or moisture-rich environments. This inherent susceptibility results in polymer leaching, deterioration in mechanical integrity, and inadequate long-term stability in aqueous environments, thereby limiting the practical applicability of such composites.

Crosslinking strategies have been extensively employed to enhance the structural integrity and functional performance of polymer systems and to stabilize hydrophilic domains within the elastomeric matrix. Ionic crosslinking using divalent cations such as Ca^2+^ is a simple and effective approach for alginate-based networks, forming egg-box junction structures that improve mechanical strength and dimensional stability [9,10]. In addition, polyphenol-mediated crosslinking using tannic acid (TA, Figure 1d) has emerged as a green and versatile strategy for constructing multifunctional polymer networks through hydrogen bonding, hydrophobic interactions, and π-π stacking. These interactions can enhance the mechanical properties, solvent resistance, and adhesive performance of resulting composites [11,12]. PVA strongly interact with TA through extensive hydrogen bonding between their hydroxyl groups, resulting in the formation of a physically crosslinked polyphenol-polymer network [13]. Such PVA-TA systems exhibit strong adhesion toward diverse substrates, including metals, ceramics, and polymers, while maintaining low cytotoxicity [14]. Despite these advantages, the incorporation of TA into hydrophilic polymer systems remains challenging. Direct mixing of PVA solution with aqueous TA solution often results in rapid gelation and precipitation. Furthermore, immersion of hydrophilic films in aqueous TA solution can cause excessive swelling and structural instability, preventing the formation of uniform freestanding films.

Previous studies have investigated the incorporation of hydrophilic polymers into deproteinized natural rubber (DPNR) latex to modify the properties of the resulting films. W. Pichayakorn et al. blended DPNR latex with several adhesive polymers, including poly(vinyl alcohol) (PVA) and sodium alginate (ALG), as separate binary blends in the presence of different plasticizers [15]. Among the polymers investigated, DPNR/PVA-based films exhibited relatively high adhesive performance and were identified as promising formulations for medical and pharmaceutical applications, whereas the incorporation of ALG resulted in limited improvements in tensile and adhesive properties. More recently, D. Sarilak et al. prepared DPNR/ALG-based composite films and ALG derivatives esterified with hydroxypropyl methylcellulose, with Ca^2+^-mediated crosslinking, to enhance water absorption and achieve controlled drug release [16]. These studies demonstrate the feasibility of incorporating hydrophilic polymers into DPNR-based systems to introduce additional functionality and highlight the potential of PVA- and ALG-containing DPNR composites for biomedical applications.

Despite these advances, natural rubber-based hydrophilic composites that simultaneously exhibit flexibility, water stability, biodegradability, and effective adhesion remain challenging to develop. In this work, flexible deproteinized natural rubber/alginate/poly(vinyl alcohol) (DPNR/ALG/PVA) films were prepared by blending DPNR latex with aqueous ALG and PVA, followed by Ca^2+^-induced ionic crosslinking and treatment in a TA-containing ethanol/water cosolvent. The cosolvent was designed to balance TA diffusion and polymer swelling, enabling uniform network formation while preserving film integrity. The combination of ALG-Ca^2+^ ionic crosslinks, and PVA-TA hydrogen-bonding networks within the elastomeric DPNR matrix was expected to improve mechanical, adhesive, and swelling properties. The resulting structure-property relationships were investigated to evaluate the films as biodegradable adhesive materials for biomedical and sustainable applications.

## 2. Materials and Methods

### 2.1. Materials

Natural rubber (NR) latex with a dry rubber content of 60 wt% and preserved with high ammonia was supplied by Chemical and Materials Co., Ltd. (Bangkok, Thailand). Deproteinized natural rubber (DPNR) was prepared according to a procedure based on Kawahara et al. [17]. Briefly, NR latex was treated with 0.1 wt% urea (RCI Labscan Limited, Bangkok, Thailand) and 1 wt% sodium dodecyl sulfate (SDS; Loba Chemie Pvt. Ltd., Mumbai, India), followed by stirring for 60 min. The mixture was then centrifuged at 9000 rpm for 30 min; the cream layer was collected and redispersed in 1 wt% SDS solution. This centrifugation and washing process was repeated twice to remove non-rubber components. The purified rubber phase was finally dispersed in 0.1 wt% SDS, and the latex was concentrated to a total solid content of 50 wt%. Poly(vinyl alcohol) (PVA; molecular weight of 13,000–23,000 g/mol, 87–89% hydrolyzed) and tannic acid (TA) were purchased from Sigma-Aldrich (St. Louis, MO, USA). Sodium alginate (ALG; viscosity 1000–1200 cP, 1% in H_2_O at 25 °C) was purchased from Lab Valley Ltd. (Bangkok, Thailand). Calcium chloride (CaCl_2_) was obtained from ANaPURE (Auckland, New Zealand). Ethanol (EtOH) was supplied by Duksan Pure Chemicals Co., Ltd. (Ansansi, Gyeonggido, Republic of Korea). Deionized water was used throughout all experiments.

### 2.2. Preparation of Deproteinized Natural Rubber/Alginate/Poly(vinyl alcohol) (DPNR/ALG/PVA) Composite Films

The DPNR latex (50 wt% solid content) was mixed with alginate (1 wt%) and poly(vinyl alcohol) (16 wt%) stock solutions according to the formulations listed in Table 1 under magnetic stirring for 60 min. Subsequently, a 0.2% *w/v* CaCl_2_ solution was slowly added at 15 wt% relative to the ALG content and the mixture was stirred for an additional 30 min to induce ionic crosslinking. The polymer compositions were selected based on preliminary formulation screening and the functional roles of the components. The preliminary screening results (Figure S1, Supplementary Materials) were used to define the concentration ranges that provided mixtures without obvious coagulation or large aggregates while maintaining DPNR as the primary elastomeric matrix. Accordingly, DPNR was fixed at 100 phr, and the con-tents of ALG and PVA were systematically varied. ALG was varied at 1, 3, and 5 phr while PVA was maintained at 40 phr. Conversely, PVA was varied at 20, 40, and 80 phr while ALG was maintained at 3 phr. The reference concentrations of 40 phr PVA and 3 phr ALG were selected based on the preliminary formulation screening, which showed suitable dispersion without excessive aggregation while preserving the DPNR-rich elastomeric matrix. The resulting dispersions were cast into silicone molds and dried at 40 °C for 24 h to obtain DPNR/ALG/PVA films. The dried films were immersed in 5% *w/v* TA solutions prepared in ethanol/water cosolvent systems. After predetermined immersion times, the films were removed, rinsed with ethanol to remove unbound TA, and dried at 40 °C for 24 h. Among the investigated compositions, DPNR/ALG3/PVA40 was used as the representative formulation for subsequent optimization of the TA-treatment conditions, including the EtOH/H_2_O ratio, immersion time, and PVA/TA ratio. This formulation was also selected for comparative characterization against ALG/PVA and PVA films to evaluate the contribution of the DPNR-based composite structure. The TA-treated films were then subjected to gel fraction analysis by water extraction and designated as DPNR/ALG/PVA-TA composite films. The films after water extraction were subsequently used for further characterization.

### 2.3. Gel Fraction Analysis

The gel fraction of the TA-treated films was evaluated by determining the insoluble fraction after water extraction. The initial dry weight (*W_i_*) of rectangular samples (15 × 15 mm) was recorded. The samples were then immersed in deionized water at room temperature for 24 h to remove the soluble fractions. After extraction, the samples were dried at 40 °C for 24 h and the final dry weight (*W_f_*) was measured. The gel fraction was calculated using the following equation:

(1) $$Gel\;fraction\;\left(\%\right)=\;\frac{W_{f}}{W_{i}}\;\times \;100.$$

### 2.4. Fourier-Transform Infrared Spectroscopy

The chemical structure and interactions of the samples were analyzed using Fourier transform infrared spectroscopy (FTIR, Tensor 27, Bruker, Billerica, MA, USA). Spectra were collected in the range of 4000–400 cm^−1^ using an attenuated total reflectance (ATR) mode at a resolution of 4 cm^−1^ and 64 scans.

### 2.5. Tensile Testing

The tensile properties of the films were evaluated using a universal testing machine (AUTOGRAPH AGX-V2, Shimadzu, Kyoto, Japan) according to ASTM D882 [18]. Film specimens were cut into rectangular strips with dimensions of 10 × 100 mm with a gauge length of 50 mm. The tensile testing was performed at a crosshead speed of 50 mm/min. Tensile strength and elongation at break were determined from the resulting stress–strain curves. At least three specimens were tested for each formulation.

### 2.6. Swelling Measurement

The swelling degree of the DPNR/ALG/PVA-TA films was determined by gravimetric analysis. Dried film specimens (20 × 20 mm) were immersed in DI water at room temperature. The initial dry weight (*W*_0_) was recorded prior to immersion. At predetermined time intervals (0–24 h), the samples were removed, gently blotted with filter paper to eliminate excess surface water, and weighed (*W_t_*). The swelling degree was calculated using the following equation:

(2) $$Swelling\;degree\;\left(\%\right)=\;\frac{{(W}_{t}-W_{0})}{W_{0}}\;\times \;100.$$

### 2.7. Adhesive Test

The adhesive properties of the films were evaluated by lap shear and T-peel tests based on modified ASTM F2255 and ASTM D1876, respectively, using a universal testing machine (AUTOGRAPH AGX-V2, Shimadzu, Kyoto, Japan) [19,20,21,22]. For lap shear test, the wet film samples (25 × 25 mm) were placed between polyurethane sheets. The T-peel test was caried out using wet films with dimensions of 25 × 180 mm by leaving free ends for gripping. To ensure intimate contact between the film and the substrates, glass plate with a 500 g weight was applied on top of the specimens. Prior to testing, the specimens were conditioned to contain 15–25 wt% water based on the dry weight of the films. All tests were performed at a crosshead speed of 50 mm/min, and at least three replicates were analyzed for each formulation.

### 2.8. Biodegradation Study in Soil Burial Test

The soil burial degradation test was conducted to assess the environmental biodegradability of the composite films. Square specimens with dimensions of 20 × 20 mm were prepared and their initial dry weights (*W_id_*) were recorded. The samples were buried in soil at a depth of 8–10 cm in plastic containers under controlled conditions (moisture content of 40–50% and temperature of 29.5 ± 0.4 °C). At predetermined time intervals (30, 90, and 180 days), the samples were carefully retrieved from the soil, gently washed with distilled water to remove adhering soil particles, and dried to constant weight at 35 °C. The final dry weight (*W_fd_*) was recorded. The weight loss percentage was calculated by percentage mass loss relative to the initial weight according to the following equation [23]:

(3) $$Weight\;loss\;\left(\%\right)=\;\frac{{(W}_{id}-W_{fd})}{W_{id}}\;\times \;100.$$

### 2.9. Morphological Characterization After Soil Burial

The surface morphology of the films before and after soil burial was examined using scanning electron microscopy (SEM, Carl Zeiss AURIGA, Thuringia, Germany). Prior to imaging, the samples were sputter-coated with gold. SEM micrographs were obtained at an accelerating voltage of 15 kV to evaluate surface erosion, and degradation-induced morphological changes.

### 2.10. Statistical Analysis

All data are presented as the mean ± standard deviation. All experiments were performed in at least triplicate. Differences among groups were analyzed by one-way analysis of variance (ANOVA) followed by Tukey’s post-hoc test; comparisons between two groups were performed using *t*-test. The effects of ALG content (1, 3, and 5 phr at 40 phr PVA) and PVA content (20, 40, and 80 phr at 3 phr ALG) on the measured properties were analyzed separately. A value of *p* < 0.05 was considered statistically significant.

## 3. Results and Discussion

### 3.1. Formation of Deproteinized Natural Rubber/Alginate/Poly(vinyl alcohol) (DPNR/ALG/PVA) Composite Films

**Figure 2:**
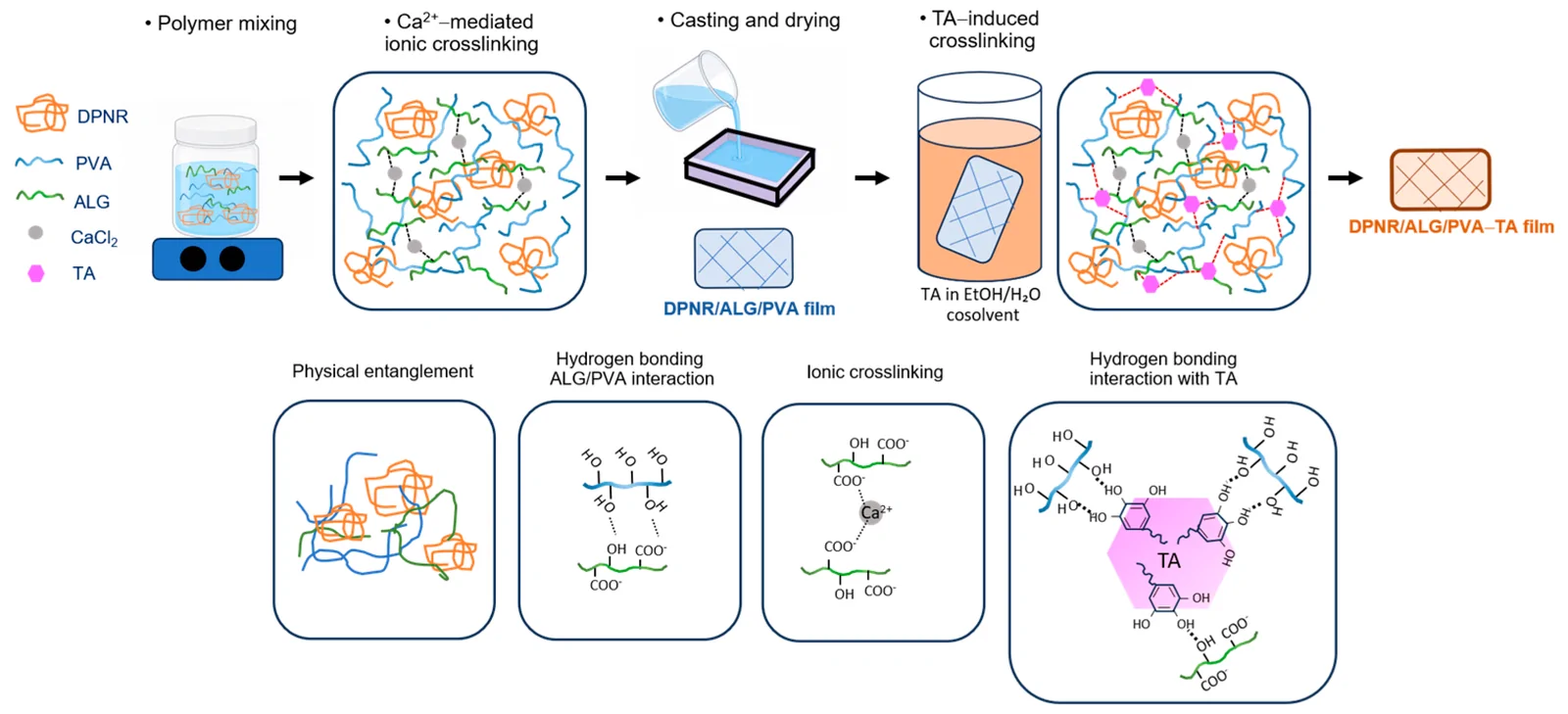
Schematic illustration of the network structure of the DPNR/ALG/PVA-TA composite film.

The bio-based composite films were fabricated through Ca^2+^-mediated ionic crosslinking followed by TA treatment, and the proposed network structure is illustrated in Figure 2. The composite structure is formed through a combination of physical entanglement among the polymer components, ionic crosslinking, and hydrogen bonding interactions. DPNR acts as a hydrophobic matrix, providing mechanical flexibility and mitigating water-induced structural deformation, while hydrophilic ALG and PVA were introduced into the DPNR latex under aqueous conditions. The DPNR latex was first stabilized with SDS, followed by the gradual addition of aqueous PVA and ALG solutions under continuous stirring. Subsequently, CaCl_2_ was added to induce ionic crosslinking between Ca^2+^ ions and the carboxylate groups (–COO^−^) of ALG. As shown in Supplementary Figure S2, no visible coagulation or large aggregates were observed during the mixing process, indicating that the DPNR/ALG/PVA dispersion remained sufficiently stable for subsequent film formation. The Ca^2+^-mediated ionic junctions within the ALG phase, together with physical entanglement among the polymer components, may contribute to a semi-interpenetrating network-like structure within the DPNR-based matrix [24]. The resulting mixtures were subsequently cast and dried to obtain the composite films. Subsequent immersion of the films in TA solutions prepared in EtOH/H_2_O cosolvent systems promoted hydrogen bonding interactions between the abundant phenolic hydroxyl groups of TA and the hydroxyl-containing groups of the polymer components, particularly PVA. These interactions may contribute to the formation of a physically crosslinked hydrogen-bonded network, which improves the cohesion of the polymer matrix and contributes to the enhanced mechanical strength and adhesive properties of the composite films [25]. The effects of solvent composition, TA content, immersion time, and polymer composition on the properties of the composite films were also examined.

### 3.2. Effect of EtOH/H_2_O Cosolvent Ratio on TA-Induced Network Formation

**Figure 3:**
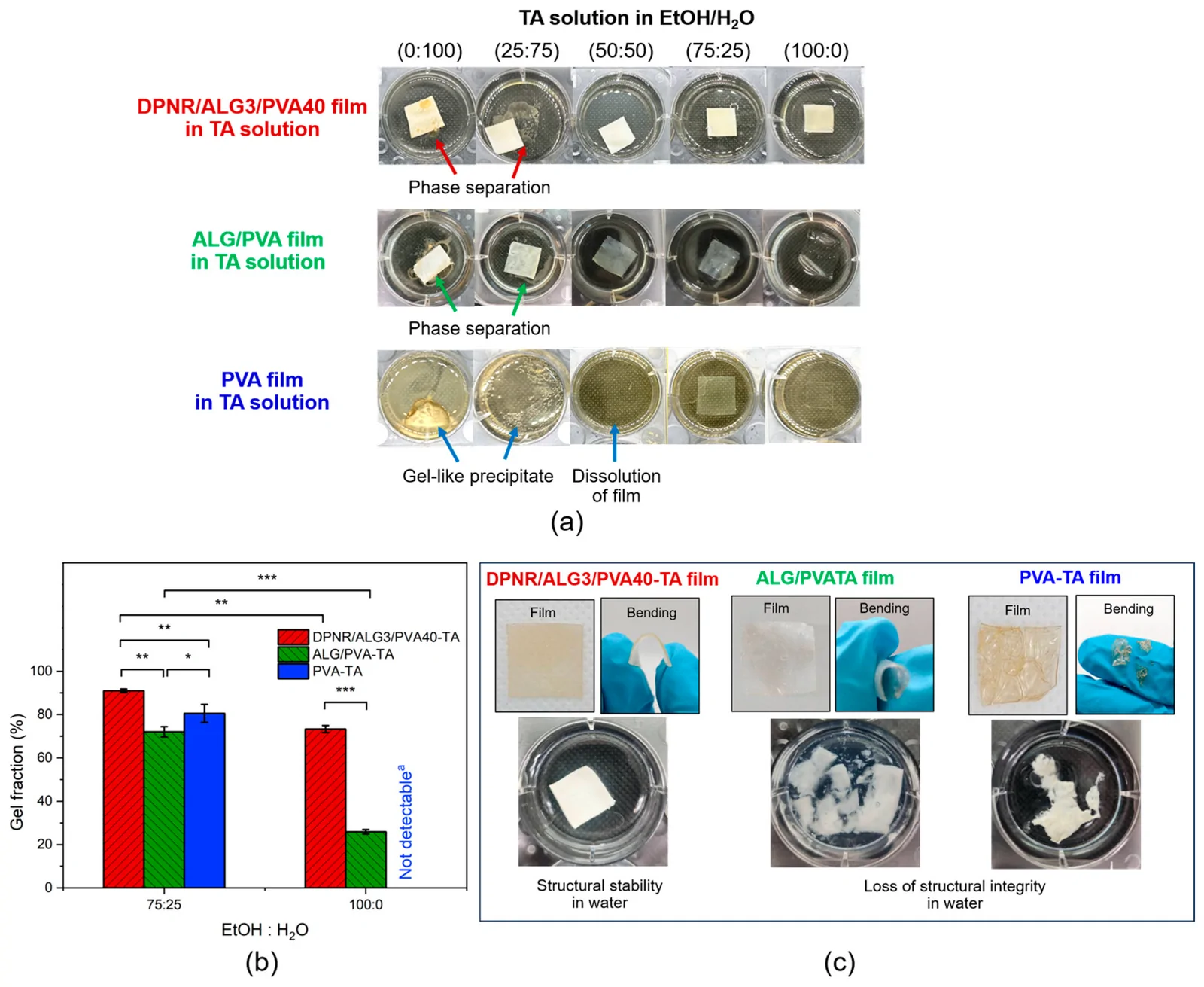
(**a**) Visual appearance of DPNR/ALG3/PVA40 films during TA treatment in EtOH/H_2_O mixed solvent systems, compared with ALG/PVA and PVA films; (**b**) Corresponding gel fraction of TA-treated films by water extraction; (**c**) Water stability and flexibility of TA-treated films during water immersion and after bending tests. (^a^ Gel fraction could not be determined due to dissolution of the sample during water extraction). Data shown as mean ± standard deviation. * *p* < 0.05, ** *p* < 0.01, *** *p* < 0.001.

The prepared films were immersed in TA solutions prepared with varying EtOH/H_2_O ratios (0:100, 25:75, 50:50, 75:25, and 100:0) to induce crosslinking through hydrogen bonding interactions. The visual appearance of the films during TA treatment is presented in Figure 3a. As shown in the figure, under H_2_O-rich conditions, partial gel-like fragments were observed to detach from the DPNR/ALG3/PVA40 film. This phenomenon can be attributed to the limited structural stability of the hydrophilic polymer components in highly aqueous environments, which promotes phase separation of the film. This behavior is more clearly illustrated by the ALG/PVA and PVA films. When immersed in H_2_O-rich media (0:100 and 25:75 EtOH/H_2_O), the ALG/PVA films exhibited gel-like material detaching from the main film. For the PVA films, they could not maintain film structure and instead transformed into precipitated gel-like materials. Such behavior can be attributed to the strong hydration of hydrophilic polymer chains in H_2_O-rich environments, which induces extensive swelling and disrupts intermolecular hydrogen bonding between polymer chains. The resulting reduction in cohesive interactions leads to structural destabilization of the film. The swollen polymer chains subsequently interact with TA, leading to the formation of gel-like precipitates. Moreover, the PVA film completely dissolved in the 50:50 EtOH/H_2_O cosolvent system. This behavior is associated with a cosolvent effect, in which polymer-solvent interactions dominate over polymer-polymer interactions, resulting in chain disentanglement and subsequent disintegration of the film [26]. Nevertheless, the DPNR/ALG3/PVA40, ALG/PVA and PVA films maintained their structural integrity in TA-containing EtOH-rich media (75:25 and 100:0 EtOH/H_2_O). Under these conditions, the film structure remains sufficiently stable to allow TA diffusion and the formation of hydrogen bonding interactions within the hydroxyl-containing polymers. The gel fraction results (Figure 3b) further demonstrate the solvent-dependent formation of the crosslinked network. The DPNR/ALG3/PVA40-TA films treated in the 75:25 EtOH/H_2_O TA solution exhibited a significantly higher gel fraction (91.01 ± 0.79%) than those treated in pure ethanol (73.29 ± 1.62%), indicating more efficient network formation in the mixed solvent. A similar trend was observed for the ALG/PVA-TA and PVA-TA films. At 75:25 EtOH/H_2_O, the gel fraction was 72.06 ± 2.40% for ALG/PVA-TA and 80.54 ± 4.13% for PVA-TA. In pure ethanol, the gel fraction of the ALG/PVA-TA film decreased significantly to 25.90 ± 1.05%, whereas the PVA-TA film dissolved completely during water extraction, so its gel fraction could not be determined. These results suggest that in pure ethanol, TA-induced hydrogen bonding interactions were predominantly localized at the film surface, leading to insufficient bulk network formation. Therefore, the 75:25 EtOH/H_2_O cosolvent was selected as the most effective of the conditions investigated, as it provided a balance among solvent-induced swelling, TA diffusion, and network stabilization, giving a physically crosslinked network with improved aqueous stability of the films. However, the resulting ALG/PVA-TA and PVA-TA films were not stable in water, as shown in Figure 3c. Moreover, the PVA-TA films became brittle and prone to fracture, indicating their limited mechanical robustness. In contrast, the DPNR/ALG3/PVA40-TA films retained both structural integrity in water and flexibility, demonstrating the key role of DPNR as a flexible reinforcing phase. The presence of hydrophobic rubber synergistically enhances the aqueous durability and mechanical resilience of the films, making them promising candidates for flexible biomedical applications.

### 3.3. Effect of TA Immersion Time

**Figure 4:**
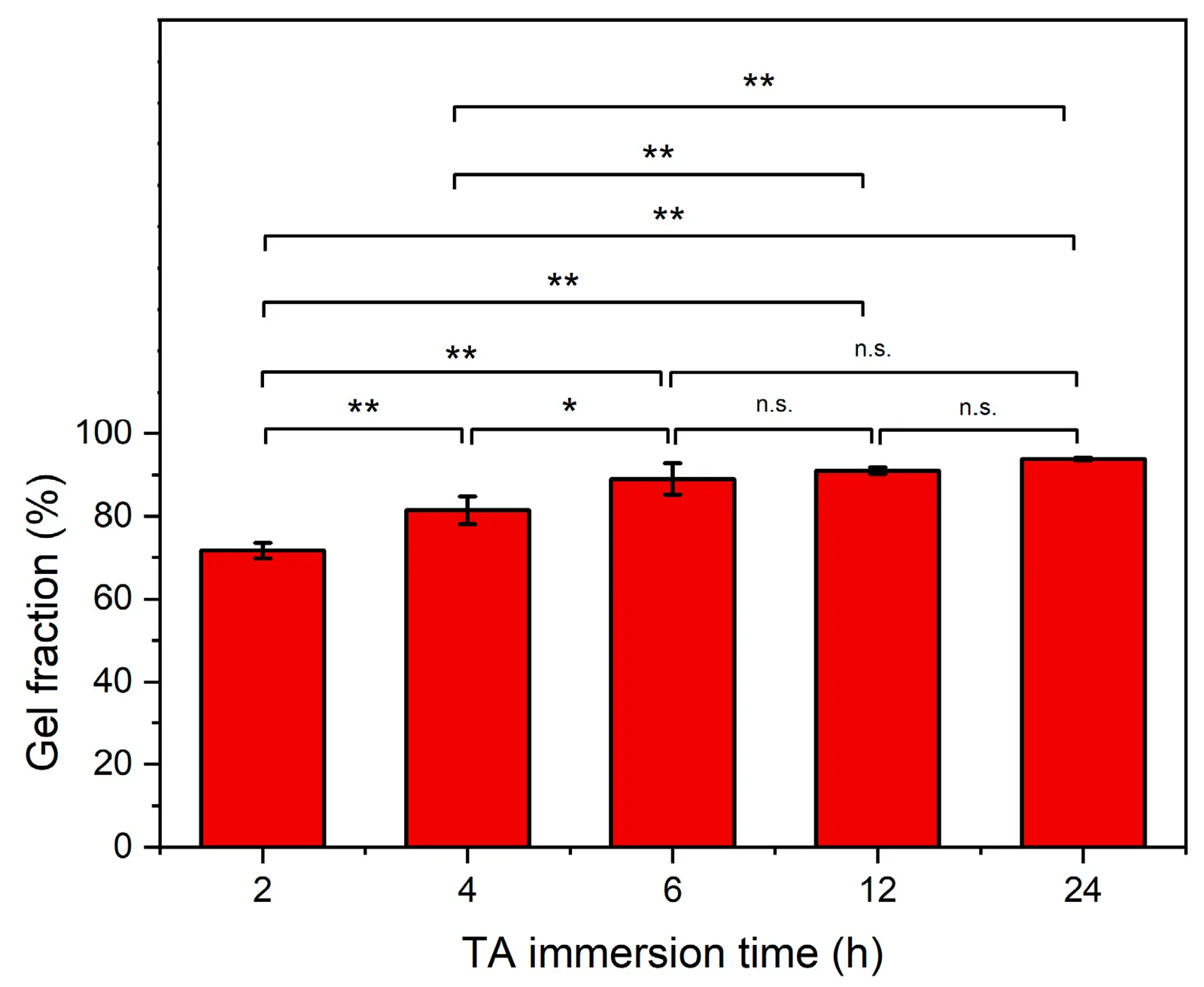
Gel fraction of DPNR/ALG3/PVA40-TA films prepared with different immersion time in TA solutions with 75:25 EtOH/H_2_O system. Data shown as mean ± standard deviation. n.s., not significant; * *p* < 0.05, ** *p* < 0.01.

Figure 4 shows the gel fraction of DPNR/ALG3/PVA40-TA films as a function of TA immersion times. The gel fraction increased significantly as the immersion time increased from 2 to 6 h, suggesting progressive diffusion of TA molecules into the interior of the film and the formation of additional hydrogen bonding interactions. At longer immersion times, the gel fraction leveled off, with no significant different, indicating that the gel fraction approached a plateau within this period. An immersion time of 12 h, at which a smaller standard deviation, was therefore used in this study, and statistical analysis confirmed that this time was within the plateau region.

### 3.4. Effect of TA Content

**Figure 5:**
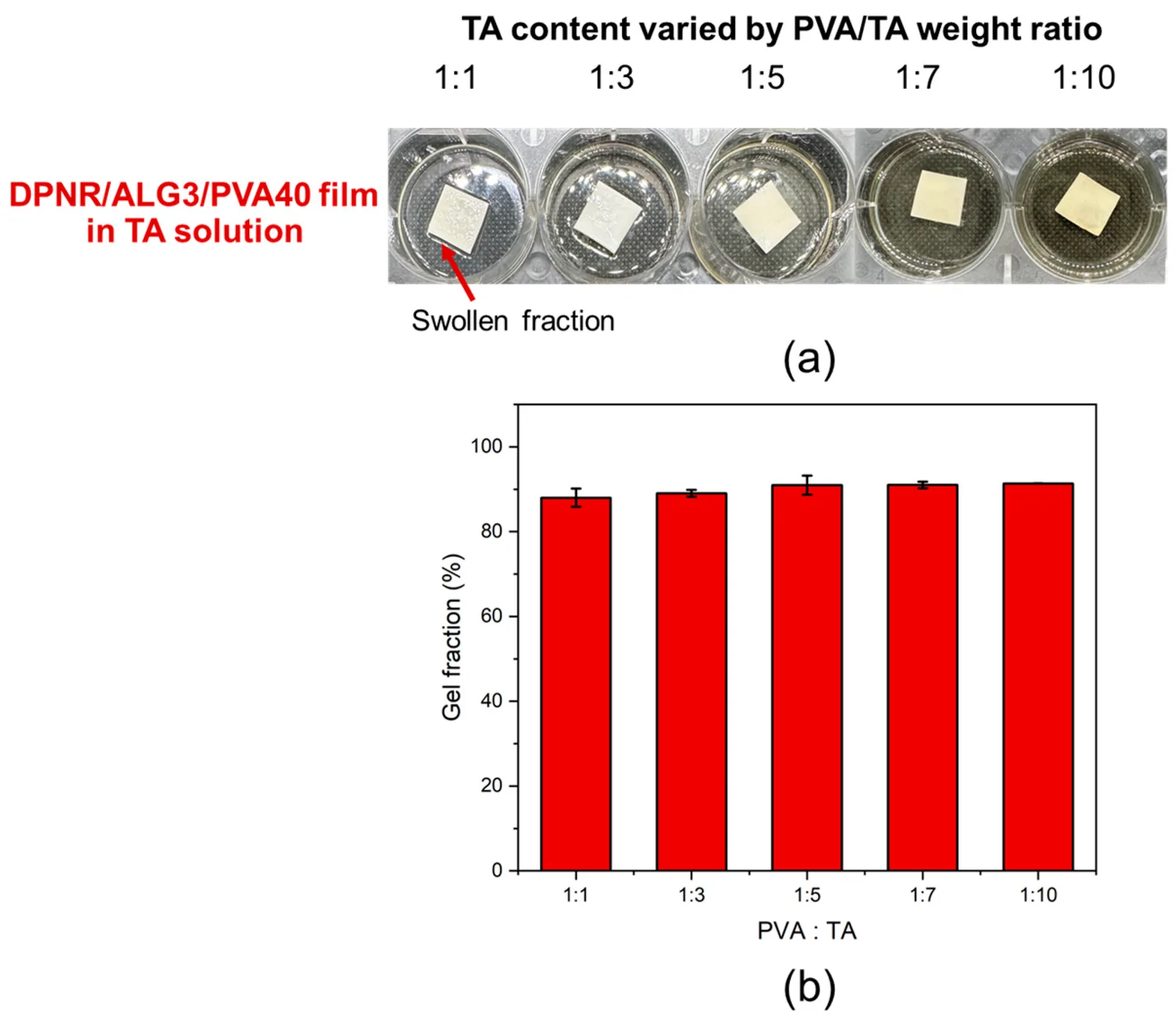
(**a**) Visual appearance of DPNR/ALG3/PVA40 films treated with varying TA contents in 75:25 EtOH/H_2_O system; (**b**) Gel fraction of TA-treated DPNR/ALG3/PVA40 films as a function of TA contents. No significant difference was found among the groups (*p* = 0.07).

The DPNR/ALG3/PVA40 films were immersed in TA solutions with varying TA contents, defined by PVA/TA weight ratios of 1:1, 1:3, 1:5, 1:7, and 1:10. As shown in Figure 5a, all films retained their macroscopic integrity across the entire range of TA contents, and the films appeared to become stiffer with increasing TA content. At a PVA/TA ratio of 1:1, swollen domains were observed protruding from the film surface, which may reflect loosely associated hydrophilic segments that were not sufficiently stabilized by TA at low TA content. The gel fraction (Figure 5b) tended to increase with TA content and appeared to level off at a PVA/TA ratio of 1:7 (91.01 ± 0.79%), suggesting extensive bulk crosslinking. However, the differences among the ratios were not statistically significant, indicating that, within the range studied, the gel fraction was not sensitive to TA content. Together with the visual observations, these results suggest that a higher TA content may help to stabilize the hydrophilic domains and stiffen the films. A PVA/TA ratio of 1:7 was therefore used in this study.

### 3.5. Effect of Polymer Composition

**Figure 6:**
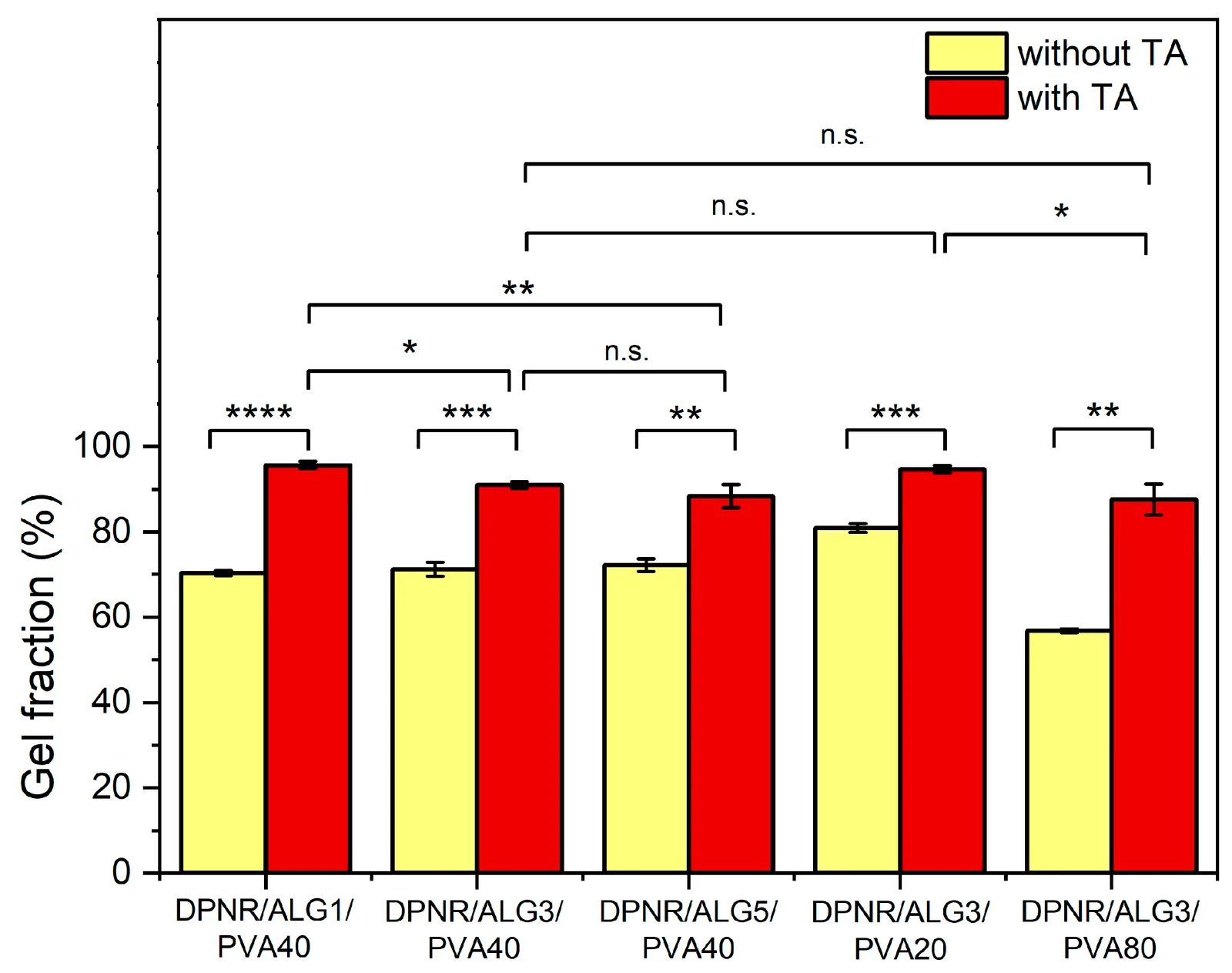
Gel fraction of the different types of DPNR/ALG/PVA composite films with and without TA treatment. Data shown as mean ± standard deviation. n.s., not significant; * *p* < 0.05, ** *p* < 0.01, *** *p* < 0.001, **** *p* < 0.0001.

The composite films were formulated by varying the ALG and PVA contents. The gel fractions of films after water extraction, with and without TA treatment, are presented in Figure 6. The influence of ALG content was investigated at 1, 3, and 5 phr with the CaCl_2_ concentration fixed at 15 wt% relative to the ALG content. A gradual decrease in gel fraction of TA-incorporated DPNR/ALG/PVA composite films was observed with increasing ALG content. This behavior can be attributed to the highly hydrophilic characteristic of ionically crosslinked ALG, which facilitates water uptake and promotes swelling of loosely associated polymer chains during water extraction. A similar reduction in gel fraction was observed with increasing PVA content (20, 40, and 80 phr). Although PVA contributes to hydrogen bonding interactions and improves film-forming capability, excessive incorporation reduces the relative proportion of the hydrophobic DPNR phase, which may render the matrix more susceptible to water penetration and swelling during extraction. In addition, the hydrophilic nature of PVA may facilitate partial dissolution of loosely bound polymer chains. Notably, the gel fraction of DPNR/ALG/PVA-TA films ranged from 87.58 ± 3.65% to 95.67 ± 0.80%, whereas the untreated films exhibited significantly lower values, ranging from approximately 56.80 ± 0.43 to 80.92 ± 1.02%, depending on the composition. These results indicate that TA treatment markedly increased the gel fraction, supporting the role of TA in establishing a physically crosslinked network.

### 3.6. Characterization of DPNR/ALG/PVA-TA Composite Films

#### 3.6.1. FTIR Analysis

**Figure 7:**
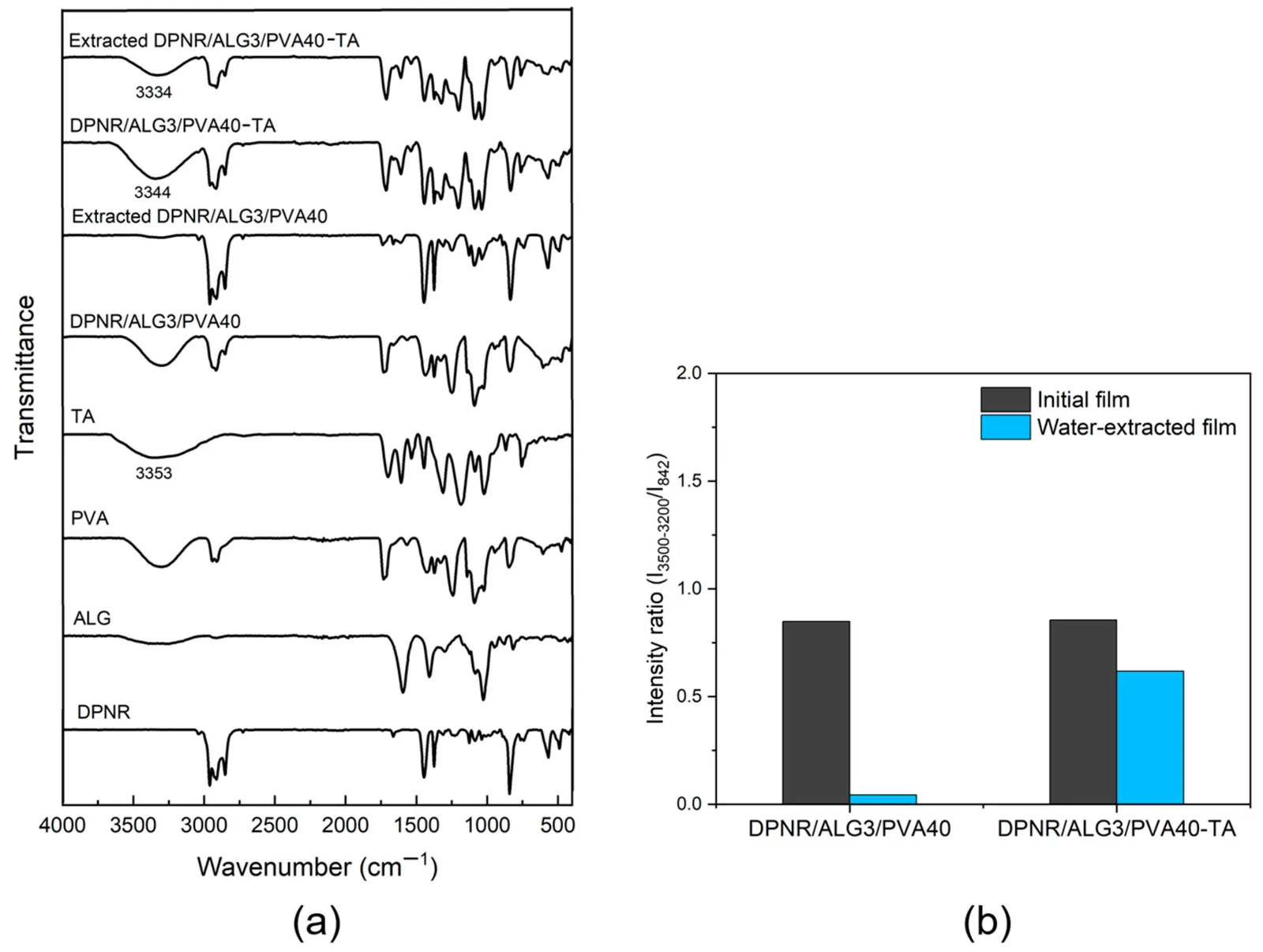
(**a**) FTIR spectra of DPNR, ALG, PVA, TA, DPNR/ALG3/PVA40 composite film, and the corresponding water-extracted composite films before and after TA treatment; (**b**) Peak intensity ratio of 3500–3200/842 cm^−1^ for the water-extracted composite films before and after TA treatment.

Figure 7a displays the FTIR spectra of DPNR, ALG, PVA, and TA, together with the DPNR/ALG/PVA films before and after TA treatment as well as the corresponding water-extracted films. DPNR exhibits characteristic absorption bands at 2961–2852 cm^−1^, corresponding to the asymmetric and symmetric stretching vibrations of aliphatic –CH groups, a peak at 1664 cm^−1^ assigned to the C=C stretching vibration of cis-1,4-polyisoprene, and peaks at 1445, 1375, and 842 cm^−1^ associated with the bending vibrations of –CH_2_–, –CH_3_, and out-of-plane bending of =C–H, respectively [27]. The absence of absorption bands related to polar functional groups confirms the nonpolar and hydrophobic nature of DPNR, supporting its role as a water-resistant matrix. Upon incorporation of ALG and PVA into the DPNR matrix (e.g., DPNR/ALG3/PVA40), a broad absorption band appears in the 3500–3200 cm^−1^ region, corresponding to O–H stretching vibrations originating from the hydroxyl-containing hydrophilic polymers [28]. The appearance of this band confirms the incorporation of ALG and PVA within the rubber matrix. After TA treatment, the DPNR/ALG3/PVA40-TA exhibited additional characteristic peaks of TA, including aromatic ring vibrations at 1609 and 1538 cm^−1^ and phenolic C–O stretching at 1202 cm^−1^ [29]. In addition, the shift in the O–H stretching vibration indicates a change in the hydrogen bonding environment of the hydroxyl groups. Together with the appearance of the characteristic TA absorption bands, this observation suggests the establishment of intermolecular interactions between TA and the hydroxyl-containing polymer chains, contributing to the formation of a TA-mediated physically crosslinked network [30]. After water extraction, a decrease in the intensity of hydroxyl-related absorption bands was observed, suggesting the removal of uncrosslinked or weakly associated hydrophilic components. To further evaluate network stability, the intensity ratio of the hydroxyl band to the DPNR reference peak (I_3500–3200_/I_842_) was analyzed as shown in Figure 7b. The water-extracted DPNR/ALG3/PVA40-TA film exhibited a higher intensity ratio than the water-extracted DPNR/ALG3/PVA40 film, indicating greater retention of hydroxyl-containing species after water extraction. This result suggests that TA treatment effectively stabilized the hydrophilic domains through hydrogen bond-mediated network formation. The FTIR findings are consistent with the gel fraction results, where TA-treated films exhibited significantly higher gel fractions than untreated films. These results demonstrate that TA functions as an effective physical crosslinker, enhancing network integrity and improving the resistance of the composite films to aqueous environments [31].

#### 3.6.2. Mechanical Property

**Figure 8:**
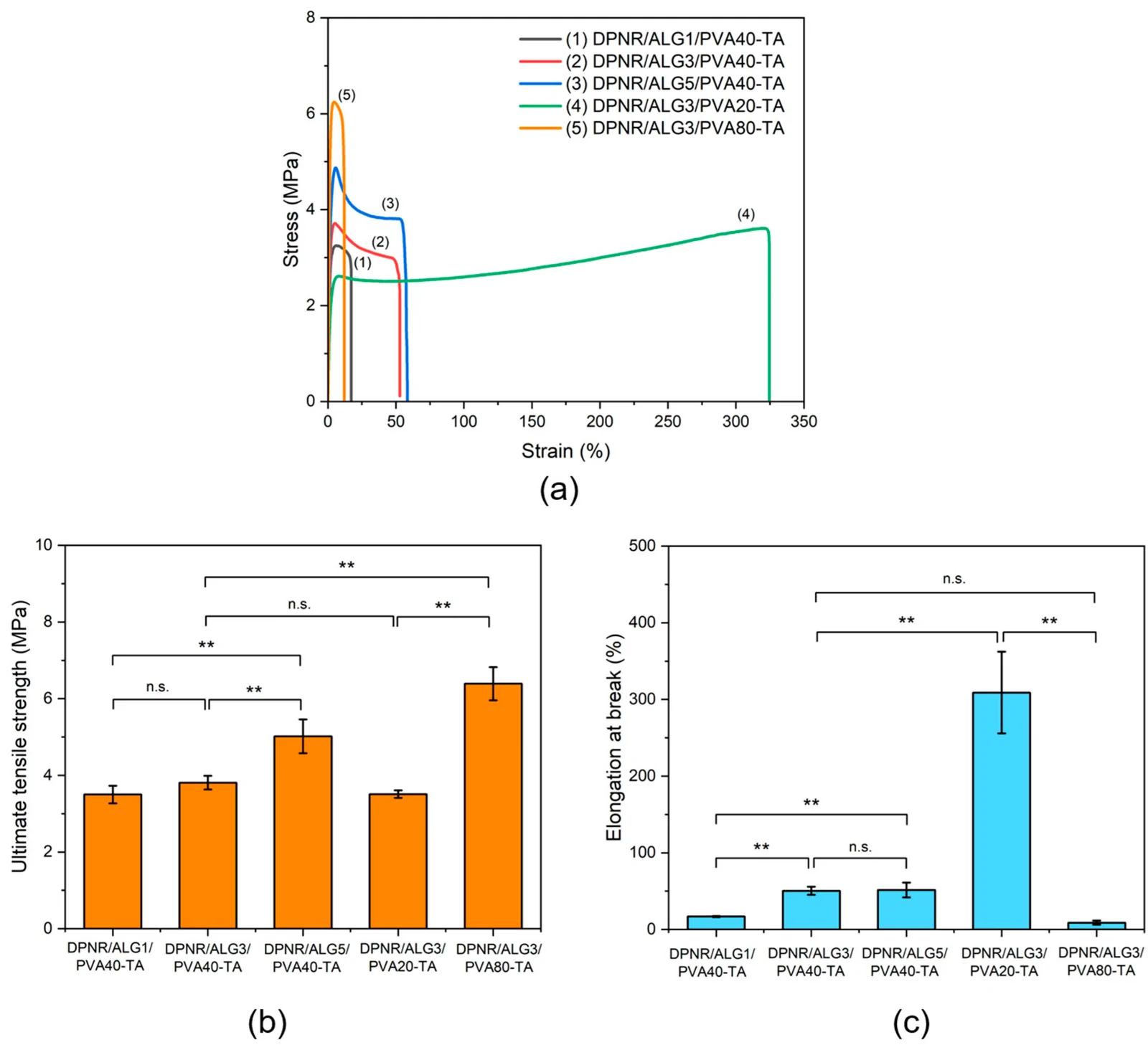
(**a**) Stress–strain curve of different types of DPNR/ALG/PVA-TA composite films; (**b**) Tensile strength; (**c**) Elongation at break of composite films. Data shown as mean ± standard deviation. n.s., not significant; ** *p* < 0.01.

The tensile properties of the DPNR/ALG/PVA-TA composite films were evaluated, and the stress–strain curves are shown in Figure 8a, with the corresponding tensile strength and elongation at break summarized in Figure 8b and Figure 8c, respectively. For the films containing 1 to 5 phr ALG, the tensile strength was comparable at 1 and 3 phr but increased significantly at 5 phr (3.50 ± 0.23 to 5.02 ± 0.44 MPa), whereas the elongation at break increased significantly from 1 to 3 phr and remained comparable between 3 and 5 phr (16.96 ± 0.78% to 51.62 ± 9.67%). These improvements can be attributed to ionic crosslinking domains formed through interactions between alginate chains and Ca^2+^ ions. The well-known “egg-box” structure generated by ALG-Ca^2+^ coordination acts as a network junction within the composite [32,33], reinforcing the composite structure while still allowing sufficient mobility of the elastomeric chains. Consequently, stress transfer efficiency within the matrix is enhanced without severely restricting chain deformation, leading to simultaneous improvements in tensile strength and extensibility [34]. As the PVA content increased from 20 to 80 phr, the tensile strength increased significantly only at 80 phr (3.51 ± 0.10 to 6.39 ± 0.43 MPa), while the elongation at break decreased significantly from 20 to 40 phr and showed no significant change between 40 and 80 phr (308.91 ± 53.32% to 8.97 ± 2.70%). This behavior is attributed to the extensive formation of intermolecular hydrogen bonding interactions between PVA chains and tannic acid molecules, which increases the effective crosslink density and promotes a denser and more rigid network structure. As a result, the composite films exhibit increased stiffness and tensile strength at the expense of extensibility [35,36]. Within the composite, the DPNR matrix acts as a flexible energy-dissipating phase, whereas the crosslinked ALG and PVA networks function as reinforcing domains, and the combination of these components is considered to contribute to the observed mechanical performance of the films.

#### 3.6.3. Swelling Degree

**Figure 9:**
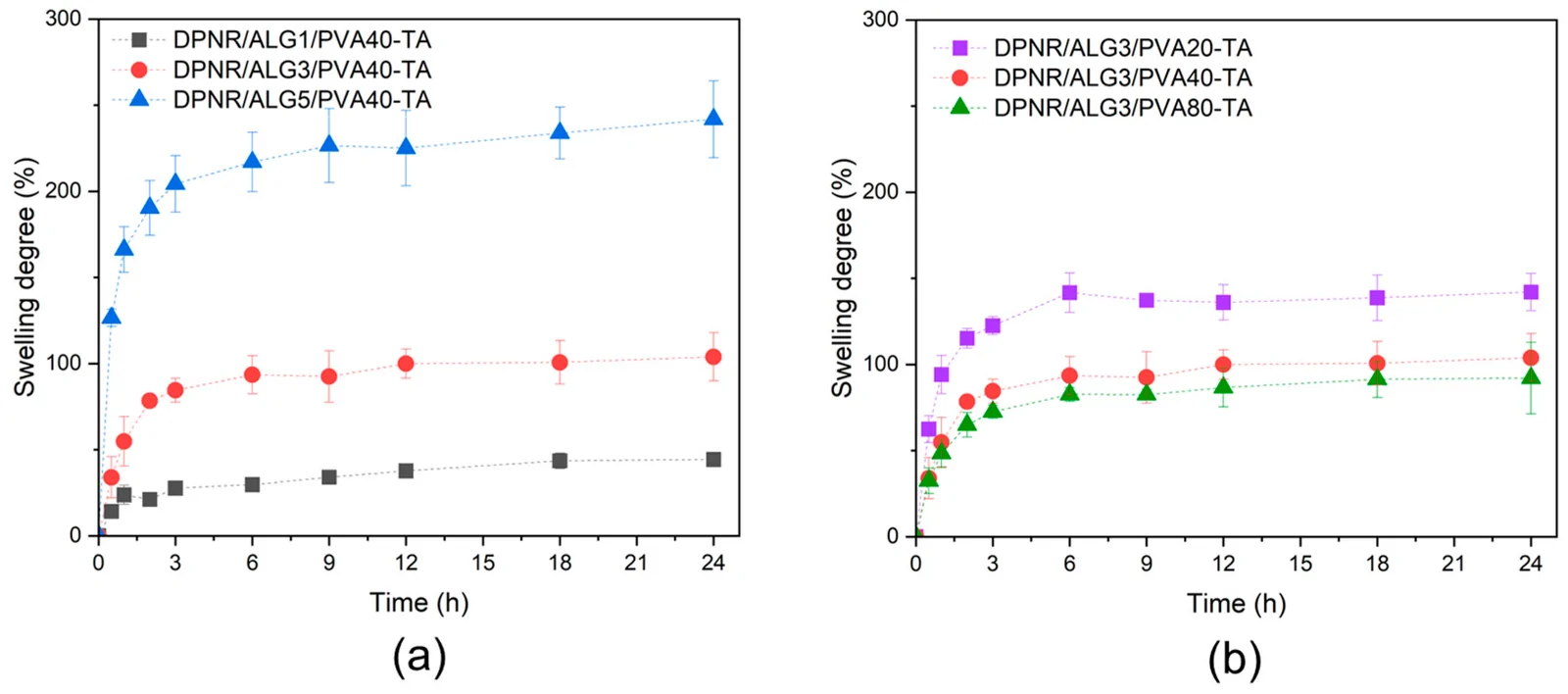
Swelling degree of DPNR/ALG/PVA-TA composite films as a function of immersion time: (**a**) Films with different ALG contents (1, 3, and 5 phr); (**b**) Films with different PVA contents (20, 40, and 80 phr).

The swelling behavior of the composite films is a crucial parameter for assessing the performance of biomedical materials, particularly wound dressings, where sufficient fluid absorption is required for effective wound exudate management while maintaining structural stability and mechanical integrity. Neat DPNR exhibited a very low swelling degree, reflecting its nonpolar and hydrophobic nature, which limits water penetration into the matrix. The incorporation of hydrophilic polymers enhanced the water uptake capacity of the composite films. For all formulations, the swelling degree progressively increased with immersion time, indicating continuous diffusion of water molecules into the composite structure until equilibrium swelling was reached. As shown in Figure 9a, increasing the ALG content enhanced the swelling capacity of the films. After 24 h of immersion, the swelling degrees were 44.35 ± 2.58%, 103.88 ± 14.01%, and 241.81 ± 22.30% for DPRN/ALG1/PVA40-TA, DPRN/ALG3/PVA40-TA, and DPRN/ALG5/PVA40-TA, respectively. This behavior can be attributed to the highly hydrophilic nature of alginate, which contains ionizable carboxylate (–COO^−^) groups along its polymer backbone. These hydrophilic functional groups promote strong interactions with water molecules and facilitate water diffusion into the composite matrix. In addition, the formation of Ca^2+^-mediated ionic crosslinks between alginate chains generates hydrophilic ionic domains within the network structure. These ionic junctions can absorb and retain large amounts of water while still maintaining network integrity. Figure 9b shows the swelling degree of DPNR/ALG/PVA-TA composites with varying PVA contents (20 to 80 phr). The swelling degree was decreased from 142.00 ± 10.83% to 92.21 ± 20.63% with increasing PVA content. This reduction can be attributed to the increased hydrogen bond density in the PVA-TA network, which increases the crosslink density and produces a dense polymer structure. As a result, polymer chain mobility and the free volume within the composite are reduced, thereby limiting water diffusion and water uptake. Similar behavior has been widely reported in hydrogel systems, where increased crosslink density reduces swelling while improving mechanical stability [37].

#### 3.6.4. Adhesive Property

**Figure 10:**
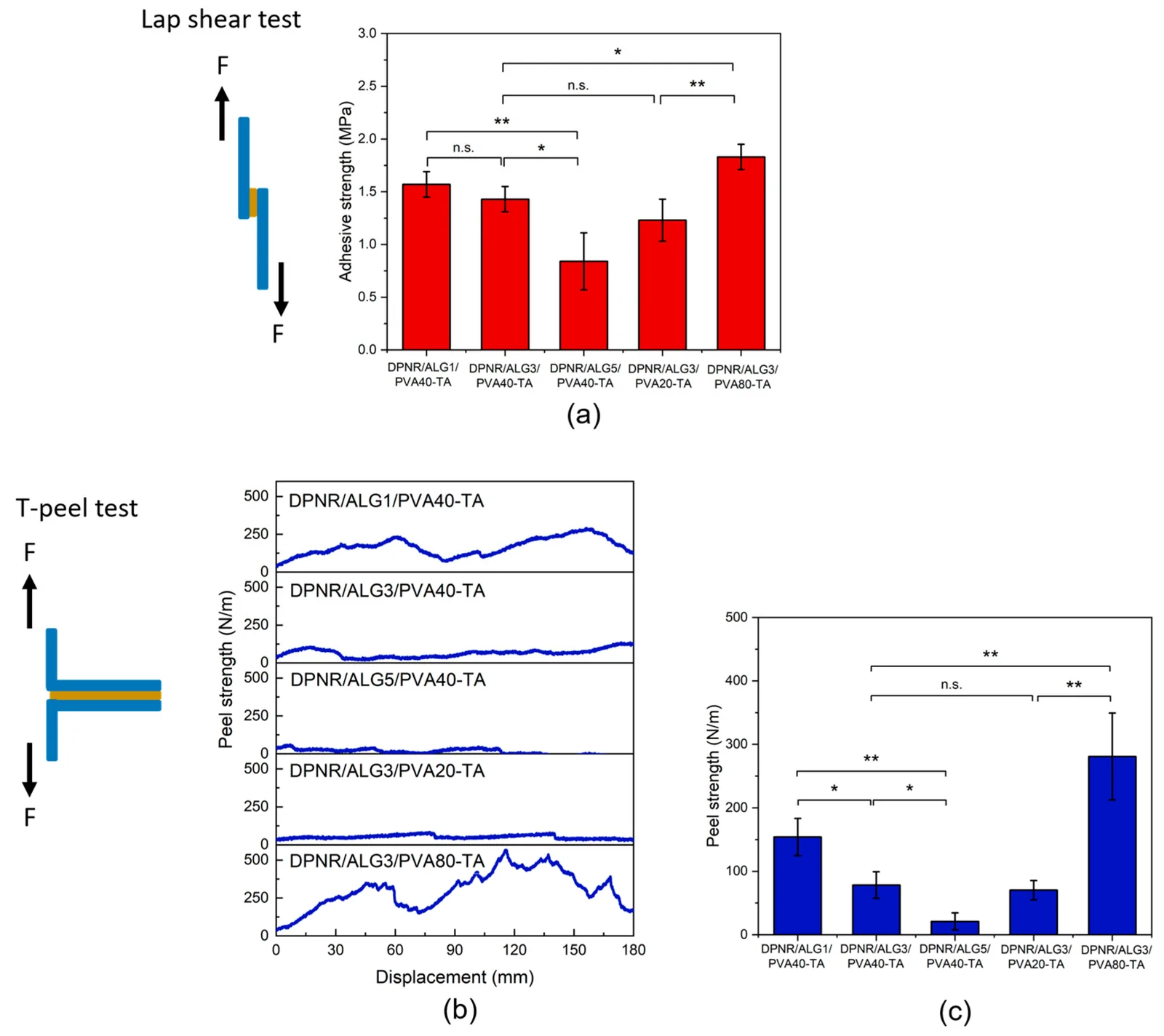
(**a**) Peel strength of DPNR/ALG/PVA-TA composite films; (**b**) Peel strength-displacement curve of the prepared DPNR/ALG/PVA-TA composite films; (**c**) Peel strength of DPNR/ALG/PVA-TA composite films. Data shown as mean ± standard deviation. n.s., not significant; * *p* < 0.05, ** *p* < 0.01.

The adhesive properties of the DPNR/ALG/PVA-TA composite films were evaluated by lap shear and T-peel tests (Figure 10). The lap shear strengths of the films containing 1, 3, and 5 phr ALG were 1.57 ± 0.12, 1.43 ± 0.12, and 0.84 ± 0.27 MPa, respectively, showing no significant difference between 1 and 3 phr but a significant decrease at 5 phr (Figure 10a). Increasing the PVA content from 20 to 40 and 80 phr gave lap shear strength of 1.23 ± 0.20, 1.43 ± 0.12, and 1.83 ± 0.12 MPa, with a significant increase only at 80 phr. The representative peel force-displacement curves are shown in Figure 10b, and the corresponding peel strengths are summarized in Figure 10c. Similar trends were observed. Increasing the ALG content from 1 to 5 phr significantly and progressively reduced the peel strength from 154.13 ± 29.17 to 21.10 ± 13.43 N/m. Increasing the PVA content from 20 to 80 phr raised it from 70.37 ± 15.10 to 280.94 ± 68.45 N/m, with a significant increase only at 80 phr. The enhanced adhesive performance is associated with the increased density of hydrogen bond-mediated interactions within the PVA-TA network [38]. The abundant hydroxyl groups of PVA interact strongly with the phenolic hydroxyl groups of TA, thereby increasing the cohesive strength of the adhesive layer. Furthermore, TA contains multiple phenolic functionalities that can establish intermolecular interactions with various functional groups present on substrate surfaces, including hydroxyl, carbonyl, and amine groups [39]. These interactions promote stronger interfacial adhesion and improve resistance to deformation. Similar adhesion-enhancing effects of polyphenol-mediated interactions have been widely reported in bioinspired adhesive systems based on tannic acid and catechol-containing compounds [40,41]. Therefore, the PVA-TA hydrogen bonded network plays a dominant role in enhancing adhesive performance through improved cohesive integrity and interfacial interactions, making the developed composite films promising candidates for wound dressing and biomedical adhesive applications.

#### 3.6.5. Soil Burial Degradation

**Figure 11:**
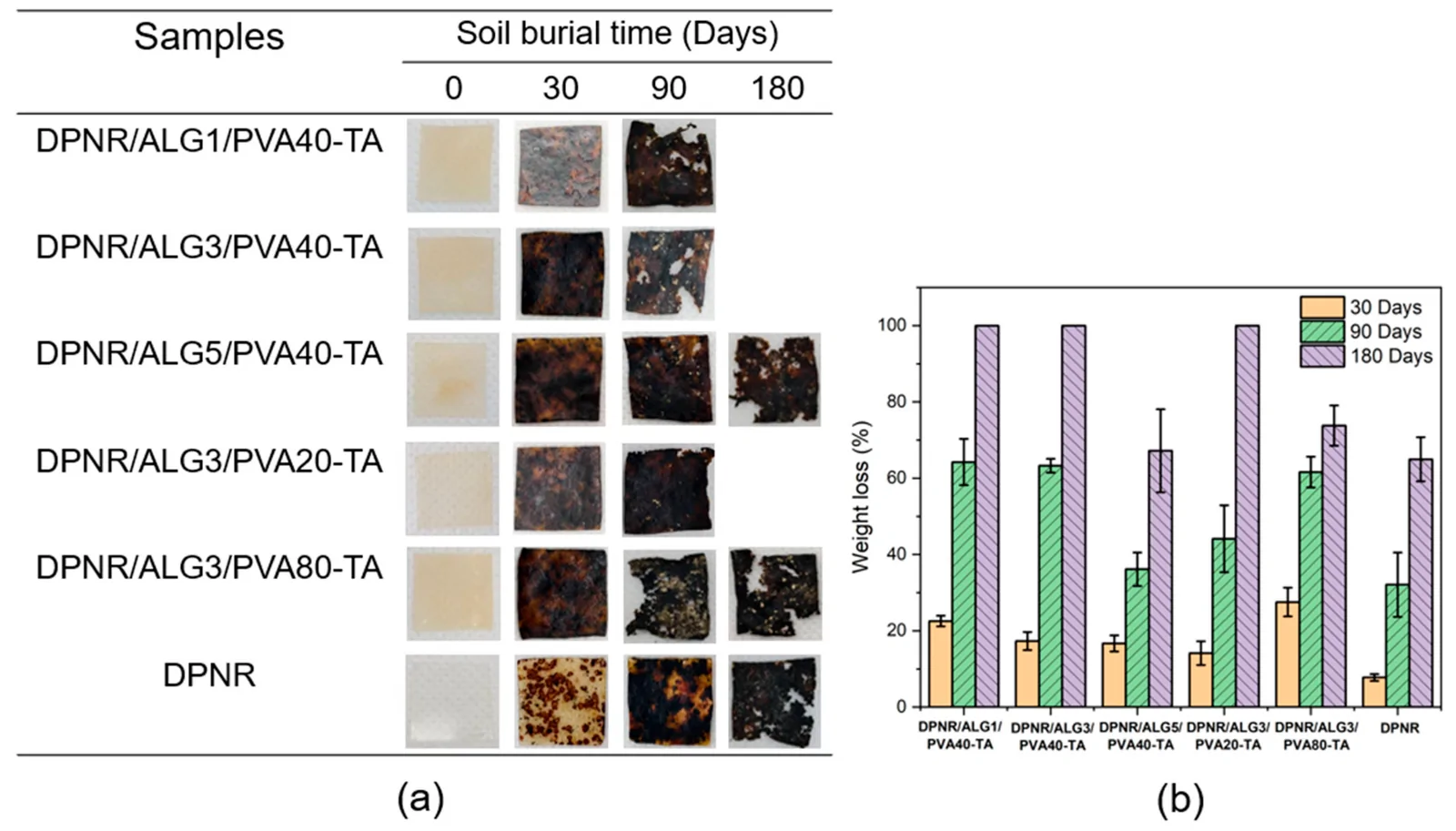
(**a**) Photographic images of the composite films before soil burial and after 30, 90, and 180 days of burial; (**b**) Corresponding weight loss percentage as a function of burial time.

The soil burial degradation behavior of the composite films was monitored over 180 days. The visual appearance of the samples before and after burial is presented in Figure 11a, and the corresponding weight loss percentages are shown in Figure 11b. After soil exposure, noticeable changes in color and physical morphology were observed, including pore formation and structural disintegration, indicating progressive biodegradation of the composites during burial. Neat DPNR exhibited the lowest degradation rate throughout the burial period, with weight loss percentages of 7.78 ± 0.91%, 32.09 ± 8.43%, and 64.97 ± 5.79% after 30, 90, and 180 days, respectively. This relatively slow degradation behavior can be attributed to the hydrophobic nature of natural rubber, which restricts water penetration and limits microbial accessibility [42]. In contrast, the incorporation of hydrophilic polymers such as ALG and PVA enhanced the degradation behavior compared with neat DPNR [43]. For the DPNR/ALG/PVA-TA composite films, the degradation behavior was strongly governed by both the polymer composition and the effective crosslink density of the network structure. The slower degradation observed at higher ALG content may be attributed to the increased density of ALG-Ca^2+^ ionic crosslinks. It was observed that the DPNR/ALG1/PVA40-TA and DPNR/ALG3/PVA40-TA films could not be recovered after 180 days of soil burial, indicating extensive degradation or fragmentation under the test conditions. However, DPNR/ALG5/PVA40-TA exhibited a mass loss of 67.21 ± 10.88%, suggesting enhanced structural stability at higher ionic crosslink density. A similar trend was observed for the DPNR/ALG/PVA-TA films with varying PVA contents. During the early stage of burial (0–90 days), DPNR/ALG3/PVA20-TA exhibited lower weight loss than DPNR/ALG3/PVA40-TA and DPNR/ALG3/PVA80-TA, which can be attributed to the higher fraction of the hydrophobic DPNR phase that limits water uptake and microbial colonization. However, after prolonged burial (180 days), both DPNR/ALG3/PVA20-TA and DPNR/ALG3/PVA40-TA became unrecoverable, whereas residual fragments of DPNR/ALG3/PVA80-TA remained detectable. The delayed degradation observed at high PVA content may also be associated with the increased crosslink density of PVA-rich regions. The formation of crystalline domains and dense PVA-TA hydrogen bonded interactions limits water diffusion and microbial accessibility within the composite. The delayed degradation at higher ALG and PVA contents is consistent with the tensile test results, which indicated the formation of denser and mechanically stronger network structures. Overall, the incorporation of hydrophilic polymers enhanced the biodegradability of DPNR by facilitating water uptake and microbial accessibility. However, increasing the density of ALG-Ca^2+^ ionic crosslinks and PVA-TA hydrogen-bonded interactions retarded degradation by restricting polymer chain mobility and reducing diffusion pathways for water and microorganisms.

#### 3.6.6. Surface Morphology After Soil Burial

**Figure 12:**
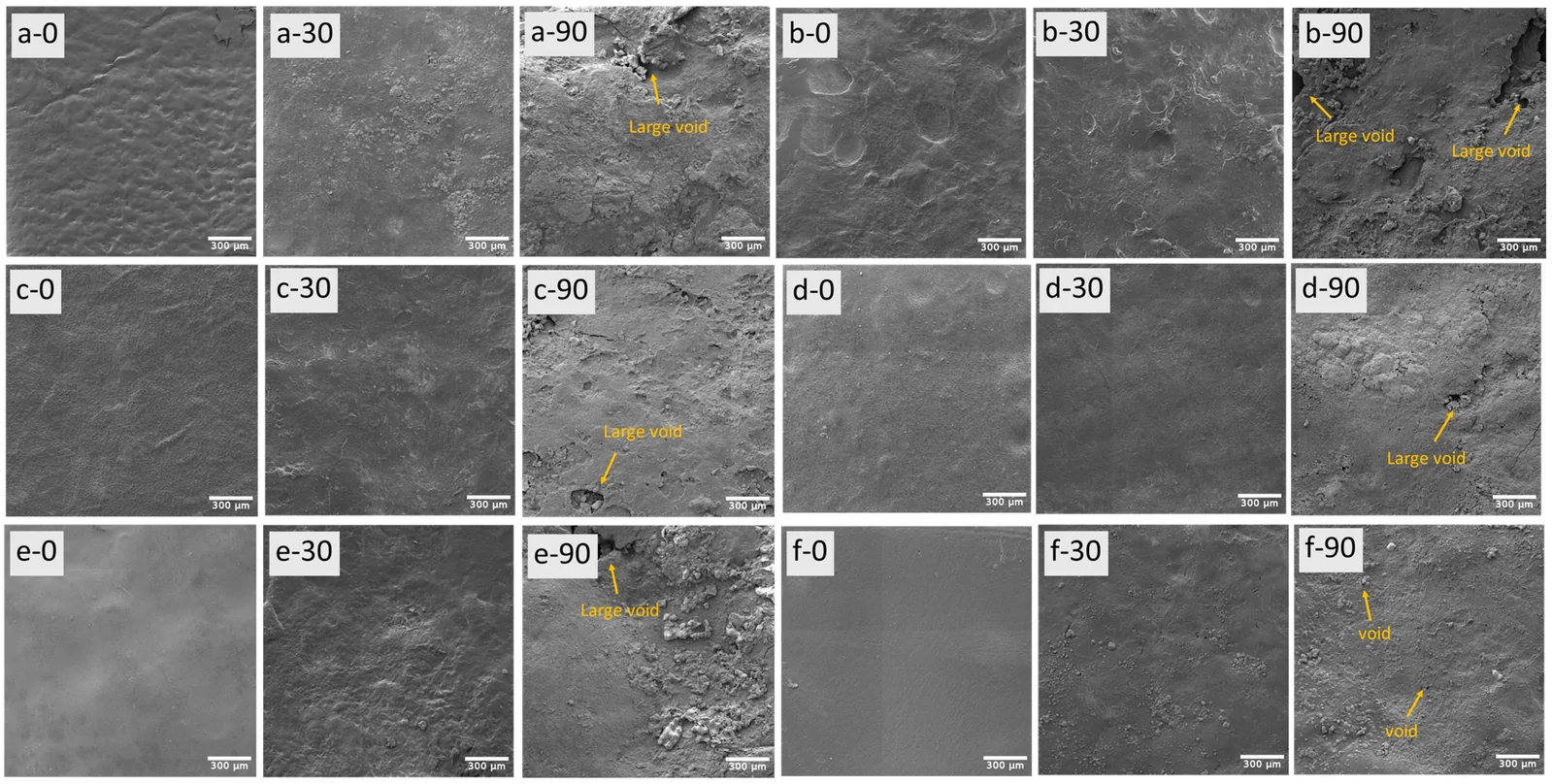
SEM images after different soil burial periods of (**a-0**,**a-30**,**a-90**) DPNR/ALG1/PVA40-TA; (**b-0**,**b-30**,**b-90**) DPNR/ALG3/PVA40-TA; (**c-0**,**c-30**,**c-90**) DPNR/ALG5/PVA40-TA; (**d-0**,**d-30**,**d-90**) DPNR/ALG3/PVA20-TA; (**e-0**,**e-30**,**e-90**) DPNR/ALG3/PVA80-TA; (**f-0**,**f-30**,**f-90**) DPNR. The numbers 0, 30, and 90 indicate the burial time (days).

The surface morphology of the composite films before and after soil burial was examined by SEM, as shown in Figure 12. Prior to burial, all samples exhibited rough but compact surfaces without visible pores. After soil burial, the surface roughness increased further and pores were formed, indicating progressive deterioration of the composite films. Neat DPNR films exhibited minor surface alterations after burial, primarily characterized by shallow surface erosion and limited roughness development. The surface remained relatively dense and intact even after prolonged burial, suggesting the resistance of the hydrophobic rubber to water penetration and microbial attack. This observation is consistent with the slow degradation rate observed in the soil burial test. Similar behavior has been reported for natural rubber, where microbial degradation is restricted by the low polarity and dense hydrocarbon backbone of the polyisoprene chains [44]. The DPNR/ALG/PVA-TA composite films exhibited morphological changes after soil burial. Progressive surface roughening and void formation were clearly observed, with the extent of structural deterioration increasing with burial time. These features suggest that degradation was initiated at the surface and gradually propagated into the bulk structure. The formation of voids may be associated with the degradation and leaching of hydrophilic ALG and PVA regions. These hydrophilic components facilitate water diffusion and microbial colonization, thereby accelerating polymer chain scission and subsequent mass loss. Similar morphological evolution has been reported in biodegradable polymer composites containing hydrophilic components, where degradation of hydrophilic phases promotes pore formation and increased structural roughness during degradation [45]. Depending on the composition and network density, the composite films exhibited different degrees of degradation, ranging from relatively limited surface changes to more extensive structural disintegration. For practical applications, the materials are required to maintain sufficient mechanical integrity and functional stability during use while remaining biodegradable after disposal, thereby minimizing their long-term environmental impact. Therefore, the prepared composite films exhibit potential as biodegradable materials for wound dressing and other environmentally sustainable applications.

## 4. Conclusions

Biodegradable DPNR/ALG/PVA-TA composite films were successfully developed by incorporating hydrophilic polymers into the DPNR matrix, combined with Ca^2+^-mediated ionic crosslinking and TA-induced hydrogen bonding interactions. The elastomeric DPNR matrix contributed flexibility and mitigated water-induced structural deformation, while ALG introduced ionic crosslinking sites that enhanced the swelling capacity and tensile strength of the composite films. In addition, the incorporation of PVA promoted hydrogen bonding interaction with tannic acid, resulting in a denser physically crosslinking network that improved mechanical strength and adhesive performance of the films. The biodegradation behavior of the composite films was influenced by the hydrophilicity and the network density. Compared with neat DPNR, the incorporation of ALG and PVA facilitated water penetration and microbial accessibility, leading to accelerated degradation and complete disintegration during 180 days of soil burial. Soil burial and SEM analyses revealed progressive surface erosion accompanied by increasing structural deterioration during degradation. The developed DPNR/ALG/PVA-TA composite films exhibited a combination of mechanical strength, adhesive capability, water absorption, and biodegradability. These properties are desirable for potential biomedical adhesive applications. This study provides the design of multifunctional natural rubber-based biocomposites for sustainable biomedical applications.

## Figures and Tables

**Table 1:** Formulations of the DPNR/ALG/PVA films.

Samples			Content (phr) *		
	DPNR/ALG1/ PVA40	DPNR/ALG3/ PVA40	DPNR/ALG5/ PVA40	DPNR/ALG3/ PVA20	DPNR/ALG3/ PVA80
DPNR	100	100	100	100	100
ALG	1	3	5	3	3
PVA	40	40	40	20	80

* Parts per hundred rubber.

## Data Availability

The original contributions presented in this study are included in the article/Supplementary Materials. Further inquiries can be directed to the corresponding author.

## Supplementary Materials

The following supporting information can be downloaded at https://www.mdpi.com/article/10.3390/polym18202536/s1: Figure S1. Preliminary formulation screening of DPNR-based polymer dispersions; Figure S2. Macroscopic appearance of the DPNR/ALG/PVA dispersions during the mixing process. No visible coagulation or large aggregates were observed after the gradual addition of aqueous ALG and PVA solutions to the SDS-stabilized DPNR latex, indicating sufficient macroscopic dispersion stability for subsequent film formation.
