# Supplementary material for: Bio-Based Deproteinized Natural Rubber/Alginate/Poly(vinyl alcohol) Films with Ca2+-Mediated and Tannic Acid-Induced Crosslinked Networks for Biodegradable Adhesive Materials
Source: Polymers (Basel). 2026 Oct 15;18(20):2536. doi: 10.3390/polym18202536 (PMC13709172; doi:10.3390/polym18202536)
Supplement: Supplementary file 1 [file polymers-18-02536-s001.zip › polymers-4612196-supplementary.pdf]

Supplementary Information

Bio-Based Deproteinized Natural Rubber/Alginate/Poly(vinyl alcohol) Films with Ca^{2+}-Mediated and Tannic Acid-Induced Crosslinked Networks for Biodegradable Adhesive Materials

Chaiwat Ruksakulpiwat [1,2], Supharat Inphonlek [1,2,3] and Yupaporn Ruksakulpiwat [1,2,*]

[1] School of Polymer Engineering, Institute of Engineering, Suranaree University of Technology, Nakhon Ratchasima 30000, Thailand

2 Research Center for Biocomposite Materials for Medical Industry and Agricultural and Food Industry, Suranaree University of Technology, Nakhon Ratchasima 30000, Thailand

3 Institute of Research and Development, Suranaree University of Technology, Nakhon Ratchasima 30000, Thailand

* Correspondence: yupa@sut.ac.th

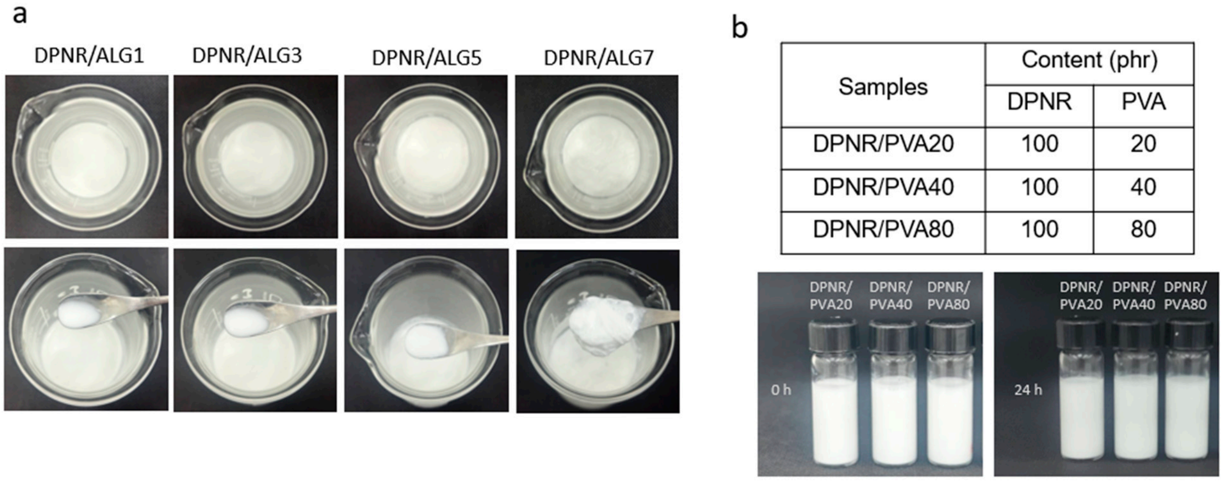

Samples	Content (phr)	
	DPNR	PVA
DPNR/PVA20	100	20
DPNR/PVA40	100	40
DPNR/PVA80	100	80

Figure S1. Preliminary formulation screening of DPNR-based polymer dispersions. (**a**) DPNR/ALG dispersions containing ALG at 1, 3, 5, and 7 phr, with $CaCl_2$ added at 15 wt% relative to the ALG content. No large aggregates were observed at ALG contents of 1, 3, and 5 phr, whereas pronounced aggregation was observed at 7 phr ALG. (**b**) DPNR/PVA dispersions containing PVA at 20, 40, and 80 phr. No visible phase separation was observed after standing for 24 h.

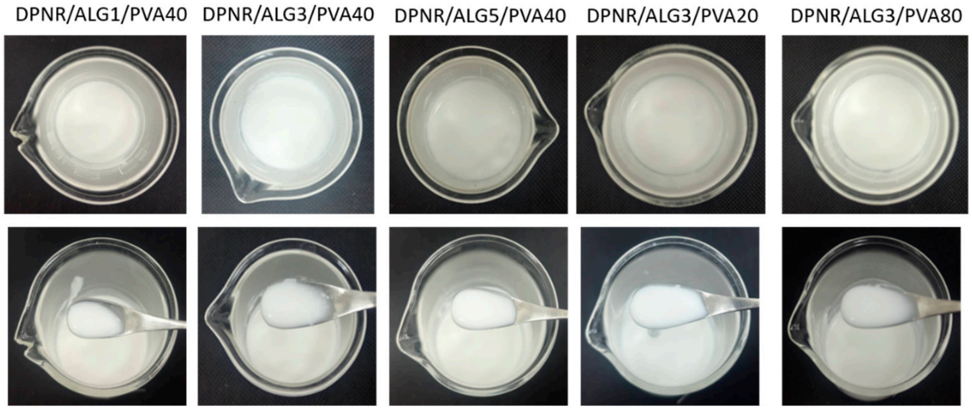

Figure S2. Macroscopic appearance of the DPNR/ALG/PVA dispersions during the mixing process. No visible coagulation or large aggregates were observed after the gradual addition of aqueous ALG and PVA solutions to the SDS-stabilized DPNR latex, indicating sufficient macroscopic dispersion stability for subsequent film formation.
